# Significant Need for a French Network of Expert Centers Enabling a Better Characterization and Management of Treatment-Resistant Depression (Fondation FondaMental)

**DOI:** 10.3389/fpsyt.2017.00244

**Published:** 2017-11-24

**Authors:** Antoine Yrondi, Djamila Bennabi, Emmanuel Haffen, Marion Garnier, Frank Bellivier, Thierry Bourgerol, Vincent Camus, Thierry D’Amato, Olivier Doumy, Frédéric Haesebaert, Jérôme Holtzmann, Christophe Lançon, Philippe Vignaud, Fanny Moliere, Isabel Nieto, Raphaëlle Marie Richieri, Philippe Domenech, Corentin Rabu, Luc Mallet, Liova Yon, Laurent Schmitt, Florian Stephan, Guillaume Vaiva, Michel Walter, Pierre-Michel Llorca, Philippe Courtet, Marion Leboyer, Wissam El-Hage, Bruno Aouizerate

**Affiliations:** ^1^Service de Psychiatrie et de Psychologie Médicale de l’adulte, Centre Expert Dépression Résistante FondaMental, CHRU de Toulouse, Hôpital Purpan, Toulouse, France; ^2^Service de Psychiatrie clinique, Centre Expert Dépression Résistante FondaMental, EA 481 Neurosciences, Université de Bourgogne Franche Comté, Besançon, France; ^3^Service de Psychiatrie clinique, Centre Expert Dépression Résistante FondaMental, Centre Investigation Clinique 1431-INSERM, EA 481 Neurosciences, Université de Bourgogne Franche Comté, Besançon, France; ^4^Service de Psychiatrie de l’adulte B, Centre Expert Dépression Résistante FondaMental, CHU de Clermont-Ferrand, Clermont-Ferrand, France; ^5^Service de Psychiatrie adulte, Centre Expert Dépression Résistante FondaMental, Hôpital Fernand-Widal, Paris, France; ^6^Service de Psychiatrie de l’adulte, CS 10217, Centre Expert Dépression Résistante FondaMental, CHU de Grenoble, Hôpital Nord, Grenoble, France; ^7^Clinique Psychiatrique Universitaire, Centre Expert Dépression Résistante FondaMental, Inserm U1253 imaging and Brain:iBrain, CHRU de Tours, Tours, France; ^8^Service Universitaire de Psychiatrie adulte, Centre Expert Dépression Résistante FondaMental, Centre Hospitalier Le Vinatier, Bron cedex, France; ^9^Pôle de Psychiatrie Générale et Universitaire, Centre Expert Dépression Résistante FondaMental, CH Charles Perrens, Bordeaux, France; ^10^Pôle Psychiatrie, Centre Expert Dépression Résistante FondaMental, CHU La Conception, Marseille, France; ^11^Département des Urgences et Post-Urgences Psychiatriques, Centre Expert Dépression Résistante FondaMental, CHRU Lapeyronie, Montpellier, France; ^12^Pôle de Psychiatrie des Hôpitaux Universitaires, Centre Expert Dépression Résistante FondaMental, Hôpital Henri Mondor, Créteil, France; ^13^Service de Psychiatrie de l’adulte, Centre Expert Dépression Résistante FondaMental, CHU de Brest, Hôpital de Bohars, Bohars, France; ^14^Service de Psychiatrie adulte, Centre Expert Dépression Résistante FondaMental, CHRU de Lille, Hôpital Fontan 1, Lille, France

**Keywords:** treatment-resistant depression, depressive disorder, assessment, network, innovative strategies

## Abstract

**Background:**

Major depression is characterized by (i) a high lifetime prevalence of 16–17% in the general population; (ii) a high frequency of treatment resistance in around 20–30% of cases; (iii) a recurrent or chronic course; (iv) a negative impact on the general functioning and quality of life; and (v) a high level of comorbidity with various psychiatric and non-psychiatric disorders, high occurrence of completed suicide, significant burden along with the personal, societal, and economic costs. In this context, there is an important need for the development of a network of expert centers for treatment-resistant depression (TRD), as performed under the leadership of the Fondation FondaMental.

**Methods:**

The principal mission of this national network is to establish a genuine prevention, screening, and diagnosis policy for TRD to offer a systematic, comprehensive, longitudinal, and multidimensional evaluation of cases. A shared electronic medical file is used referring to a common exhaustive and standardized set of assessment tools exploring psychiatric, non-psychiatric, metabolic, biological, and cognitive dimensions of TRD. This is paralleled by a medico-economic evaluation to examine the global economic burden of the disease and related health-care resource utilization. In addition, an integrated biobank has been built by the collection of serum and DNA samples for the measurement of several biomarkers that could further be associated with the treatment resistance in the recruited depressed patients. A French observational long-term follow-up cohort study is currently in progress enabling the extensive assessment of resistant depressed patients. In those unresponsive cases, each expert center proposes relevant therapeutic options that are classically aligned to the international guidelines referring to recognized scientific societies.

**Discussion:**

This approach is expected to improve the overall clinical assessments and to provide evidence-based information to those clinicians most closely involved in the management of TRD thereby facilitating treatment decisions and choice in everyday clinical practice. This could contribute to significantly improve the poor prognosis, the relapsing course, daily functioning and heavy burden of TRD. Moreover, the newly created French network of expert centers for TRD will be particularly helpful for a better characterization of sociodemographic, clinical, neuropsychological, and biological markers of treatment resistance required for the further development of personalized therapeutic strategies in TRD.

## Introduction

Major depression is characterized by a high lifetime prevalence of 16–17% in the general population (1) with the possibility of evolution into a recurrent or chronic course (2) and a negative impact on the general functioning and quality of life (3). Major depression also has a high level of comorbidity with various psychiatric and non-psychiatric disorders (1, 4), with a high occurrence of completed suicide (4), and significant burden associated with the heavy personal, societal, and economic costs (4). Treatment-resistant depression (TRD) has been estimated to represent half of the overall treatment costs for major depression (5, 6). Although not clearly established in France, it can be assumed that approximately 20–30% of depressed patients experience TRD, as reported in Anglo-Saxon countries (7), and up to one half of patients responding only partially (8). TRD is currently defined by the failure of at least two attempts of antidepressant treatments administered sequentially at adequate dose and duration (8). Patients may be unresponsive to usual therapeutic strategies because of clinical features of the condition itself, factors interfering with the proper delivery of an optimal treatment such as under-dosing or poor adherence related to side effects, cognitions, comorbid disorders (psychiatric and non-psychiatric), etc. (5, 6, 8–12). Although controversial (13), one of the major issue relies on the misdiagnosis of TRD instead of BP-II because hypomanic symptoms are difficult to detect, especially on a retrospective basis (14). Indeed, as reported by Judd et al. (15), patients with BP-II were symptomatic more than half of all follow-up weeks while depressive symptoms were largely more preponderant during the course as compared with hypomanic and cycling/mixed symptoms. Unrecognized bipolar spectrum illness increases substantially the risk for the inappropriate prescription of antidepressant treatment known to be associated with the occurrence of short-term resistance, poor tolerance, cycle acceleration, manic switch as well as late response loss after several months of recovery (13, 14, 16, 17). Thus, an inaccurate diagnosis almost always means a significant delay in appropriate and effective treatment based on mood stabilizers for bipolar patients. However, in this study, the adopted procedure tends to minimize hidden bipolar disorders behind TRD cases by using the structured Mini International Neuropsychiatric Interview (MINI) for the diagnostic evaluations coupled with the Mood Disorder Questionnaire (MDQ) useful for screening signs and symptoms of bipolar spectrum disorders.

Also, there is a heterogeneous etiology of TRD, including different environmental risk factors like childhood adversities coupled with multiple genetic determinants related to numerous genetic loci, and various epigenetic contributors involving a wide range of biological systems including the hypothalamic–pituitary–adrenal axis, immune function, monoamines, neurotrophic factors, etc. (5, 6, 8, 9, 18). These factors can significantly modulate the therapeutic response while remission remains the aim of treatment for depression, given its implications for recovery of daily functioning and better long-term prognosis (7, 19–22). The absence of appropriate treatment increases the burden of depression and the resulting costs. This burden manifests itself in many ways including residual symptoms, cognitive impairment, relapse and recurrence, decreased quality of life, suicide, cardio- and cerebrovascular morbimortality, psychosocial alteration, work days lost, family impact, and economic costs (4, 23). Taken together, all these considerations underlie the particular relevance for the determination of contributing factors by a large and exhaustive assessment of patients suffering from TRD.

Many recommendations for clinical practice have been developed by various psychiatric societies around the world (24–28). These recommendations are primarily based on the analysis and classification of the scientific literature. They consider randomized, controlled and double-blind studies on large samples as offering the highest level of evidence. In spite of their rigor from a methodological and scientific point of view, the adaptability of these recommendations in everyday clinical practice can raise questions (29). High-level evidence studies, in fact, refer to selected samples of subjects, often free from comorbidities. They exclude characteristics such as advanced age, increased suicidal risk or high level of pharmacological resistance. These clinical situations are, however, frequently observed in daily clinical practice in the patient population suffering from TRD (12, 30).

In France, the need for improving the diagnosis, assessment, and management of psychiatric pathologies has led both the Ministry of Research and the Ministry of Health to support jointly the development of a national network of expert centers for Bipolar disorder (31), schizophrenia, TRD, and autism under the leadership of the foundation of scientific cooperation named “FondaMental” created in 2007. This article describes a new model of clinical collaboration between the expert centers and the regional community of clinicians, especially those general practitioners and psychiatrists who provide the first point of contact with health services for most TRD patients. Thus, within this network, we propose a systematic, comprehensive, longitudinal, and multidimensional assessment for patients who have failed to achieve remission after at least two adequate trials using different classes of antidepressants. The psychiatric, non-psychiatric, socioenvironmental, metabolic, biological, and cognitive dimensions of the current depressive episode are explored. A personalized evaluation and care program is delivered by a clinical expert highly specialized in the field of TRD. It also intends to narrow the gap between research knowledge and clinical practice. This represents an additional and locally accessible approach to the patients and their referent physician. The objective of this article is to disseminate information about this highly relevant project and to describe the standardized evaluation as proposed within the French network of centers specifically dedicated to TRD.

## Methods

### Mission of the National Expert Centers for TRD

The principal mission of this national network is to establish a genuine prevention, screening, and diagnosis policy for TRD:

- Care: setting up a national and European network of expert centers—pluridisciplinary diagnosis and screening platforms—making it possible to follow patient cohorts with the contribution of an individual computerized medical file system common to all centers (e-Resistant Depression). This should allow access to personalized medicine primarily based on the elaboration of a strategy of care consistent with available data of the Evidence-Based Medicine and the monitoring of clinical outcomes via repeated assessments over time.
- Research: bringing together a national network of public and private researchers, linked to fundamental research platforms (mainly referring to biochemistry, molecular biology, genetic, and brain imaging), an approach that has already led to major discoveries. One of the aims is to improve staging and evaluation of innovative therapeutic options according to new scientific hypotheses, such as the inflammation theory of major depression and treatment failure (32).
- Training health-care professionals and raising awareness in the workplace: disseminating knowledge about the risk factors for mental illnesses and about novel treatment strategies.
- Communication: changing the way in which psychiatric diseases are generally perceived by the general population, and by opinion leaders, to substantially decrease stigmatization.

**Figure 1:**
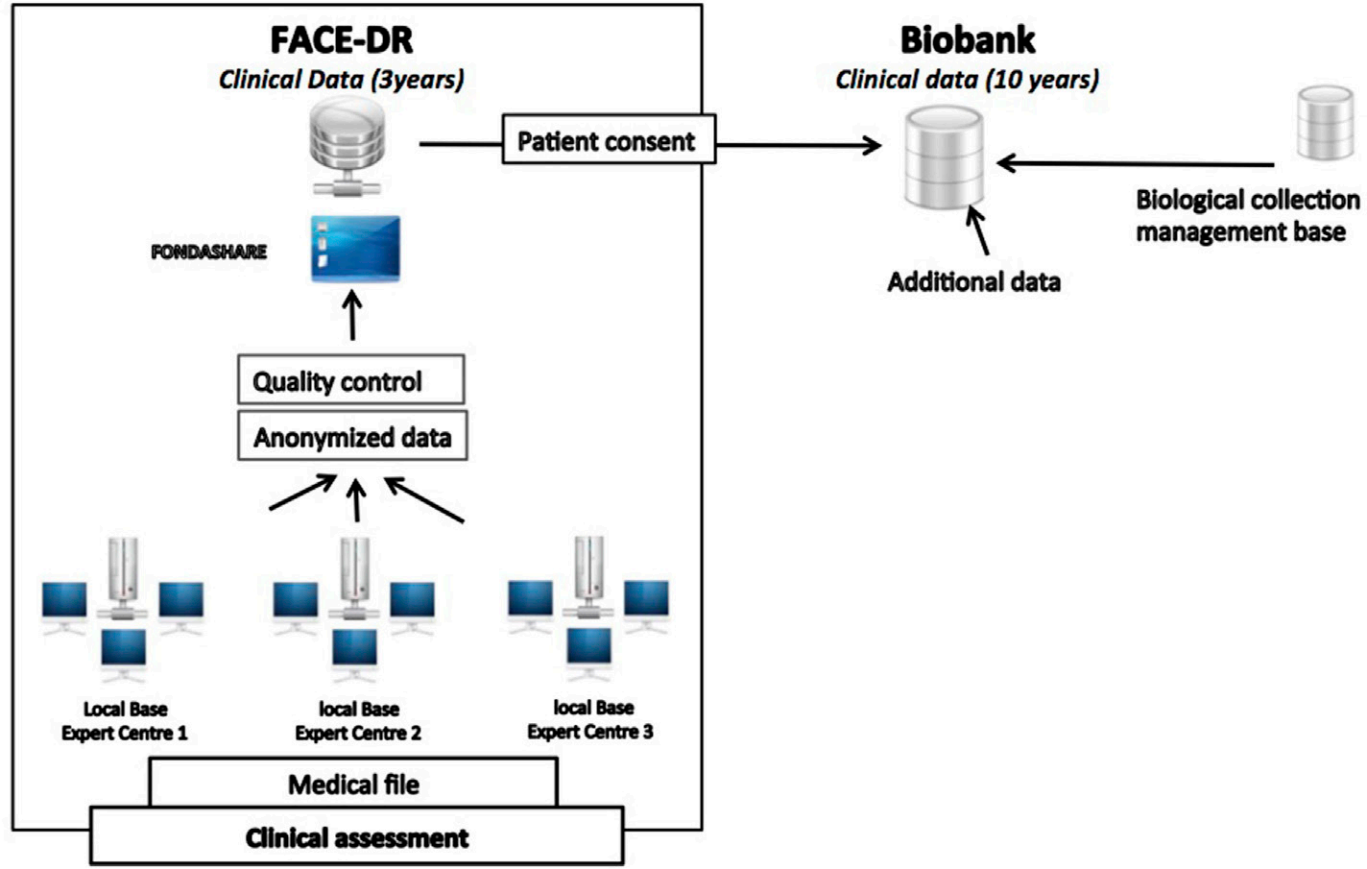
Treatment-Resistant Depression Network organization throughout the French territory.

This operating care system offers a systematic, comprehensive, longitudinal, and multidimensional evaluation of cases of TRD by sharing a common set of assessment tools exploring separately psychiatric, non-psychiatric, metabolic, biological, genetic and cognitive dimensions of major depression. This is paralleled by a medico-economic evaluation for examining the global economic burden of the disease and related health-care resource utilization. For this purpose, the shared e-resistant depression medical file (e-Resistant Depression) and database (FACE-DR) were developed by the Fondation FondaMental to collect and store all these data. In addition, an integrated biobank intimately connected to FACE-DR is now being established with the primary goal of the collection of serum, plasma, DNA, RNA samples for the measurement of several biomarkers that could further be associated with treatment resistance in depressed patients (Figure 1).

### Network of Centers

The French network of expert centers for resistant depression consists of 13 specialized care centers hosted within academic departments of Psychiatry widely distributed across France (Paris/Créteil, Paris Fernand-Widal, Besançon, Bordeaux, Brest, Clermont-Ferrand, Grenoble, Marseille, Montpellier, Lille, Lyon, Toulouse, and Tours). A multidisciplinary team of clinical representatives from FondaMental formed a working group to select the appropriate instruments for the clinical and biological assessments. These teams are composed of, at least, a psychiatrist, a nurse, a neuropsychologist and a secretary within each center. Valid and reliable observer- and self-rating scales were prioritized, and all those involved in patient assessments received intensive training in the use of the measures they employ.

### Cohort of Patients

A general practitioner or psychiatrist, who afterward will receive a detailed evaluation report along with some suggestions for clinical issues and therapeutic interventions, refers patients to be assessed in expert centers. Patients are clinically unresponsive to two successive and adequate sequences of antidepressants from two different pharmacological classes corresponding to at least stage II of the staging criteria proposed by Thase for defining TRD (33).

Before participating in the full assessment, patients are interviewed by a psychiatrist at the expert center to:

– Confirm the diagnosis of TRD according to the DSM-IV (34) criteria with moderate to severe symptoms (MADRS ≥ 20), the level of resistance indicated by the classification of Thase and Rush (33) (≥2), and assess the need to perform the full evaluation.
– Exclude bipolar disorders, psychotic disorders, OCD, eating disorders (with BMI < 15), somatoform disorders, and mood disorders related to substance abuse or misuse.
– Inform the patient about the formal assessment procedure and schedule the appointments with different health professionals within the expert centers comprising at least a psychiatrist, a nurse, and a neuropsychologist. Patients with TRD are then invited to participate and complete the assessment protocol over a period of two consecutive days.

### Data Collection

An electronic health-care record system for a common national database was implemented through the development of a web-based application, e-resistant depression© designed to collate all assessment data for clinical monitoring, research, and treatment purposes. Access to the system is carefully regulated and approval was obtained from the ethical committee and the committee in charge of the safety of computerized databases (French CNIL: DR-2015-673). To optimize data entry and retrieval, free text input has been minimized, and drops down lists and other approaches leading to standardized inputs chosen when possible. The XML format is used to transfer data from e-resistant-depression© into an anonymous national database.

### Assessment Procedure

Patients benefit at inclusion from an exhaustive clinical assessment (e-Resistant Depression) followed by systematic evaluations during four consecutive years, which are designed to identify: (i) clinical characteristics of resistance with psychological, non-psychiatric and metabolic data, (ii) socioenvironmental factors including daily routines, sport exercise, diet and stress-related factors such as early life traumatic experiences conferring high sensitivity to subsequent negative events over the lifespan which is expected to further influence the course of major depression and therapeutic response, and (iii) economic impact assessed by several indicators including number and duration of hospitalizations, number of visits to psychiatrists, general practitioners, pharmacological and non-pharmacological treatment consumption, work productivity, and health-related quality of life.

For this purpose, all centers have adopted the same package of evaluations and the different members of the center perform the full assessment. Table 1 provides a list of the self- and observer-rated measures included at each visit performed over two consecutive days.

**Table 1:** Instruments.

Description	Rater-administered instruments	Self-administered instruments
Depression severity	MADRS	QIDS-SR16
Self-esteem		Rosenberg
Manic severity	YMRS	
Manic polarity		MDQ, Altman
Anxiety severity	BAS	STAI-A
Emotional reactivity		MATHYS
Impulsivity		Barratt
Suicide	ISF, C-SSRS, SIS	
Overall severity/improvement	CGI-S/I	
General functioning	FAST/GAF	
Personality traits	BFI	
If comorbid social phobia	SPIN	
Traumatic events	PCL-S	CTQ
Stressful life events and impact	Paykel/Amiel-Lebigre	
Type/quantities care needs and resources	CSRI	
Side effects		Prise M
Therapeutic adherence		MARS
Work productivity and absence	LEAPS	
Diet		Diet questionnaire
Physical activity		Physical activity questionnaire
Physical/mental health		EQ-5D-5L
Sleep quality, sleep/awake cycle, drowsiness		PSQI/CSM/Epworth

*CSM, composite scale of morningness; EQ-5D-5L, EuroQol Five Dimension Five Level; FAST, Functional Analysis Screening Tool; GAF, Global Assessment of Functioning; MDQ, Mood Disorder Questionnaire; PSQI, The Pittsburgh Sleep Quality Index; QIDS-SR16, Quick Inventory of Depressive Symptomatology self report; SIS, Suicide Intent Scale; SPIN, Social Phobia Inventory; STAI-A, State-Trait Anxiety Inventory A; YMRS, Young Mania Rating Scale; BFI, Big Five Inventory; PCL-S, Post Traumatic Stress Disorder Check List Scale; CGI-S/I; Clinical Global Impression Severity/Improvement; C-SSRS, Columbia-Suicide Severity Rating Scale; CSRI, Client Service Receipt Inventory; CTQ, Child Trauma Questionnaire; ISF, Measure of Suicidal Ideation; LEAPS, Lam Employment Absence and Productivity Scale; MARS, Medication Adherence Rating Scale*.

For each patient, the members of the center collect sociodemographic characteristics as well as clinical data related to the assessment of symptom severity, overall functional and quality-of-life impairment (MADRS, Young Mania Rating Scale, Functional Analysis Screening Tool, Global Assessment of Functioning, and EuroQol Five Dimension Five Level) (35–39), past and present history of major depression (MINI), psychiatric and somatic comorbidities, identification of previous and current pharmacological treatments (ATHF) (40) with adverse effects (Patient Rated Inventory of Side Effects) (41) and compliance (Medication Adherence Rating Scale) (42), determination of circadian typology (The Pittsburgh Sleep Quality Index, composite scale of morningness, and Epworth) (43–45), occurrence of suicidal events (ISF, Suicide Intent Scale, Columbia-Suicide Severity Rating Scale) (46–48), along with social, environmental and medico-economic data examining childhood trauma exposure (Child Trauma Questionnaire) (49) and stressful life events (Paykel) (50), physical exercise practice (International Physical Activity Questionnaire) (51), dietary habits with the daily caloric intake, functioning at work (Lam Employment Absence and Productivity Scale) (52) and patient’s use of medical health and social care services (Client Service Receipt Inventory) (53) (Table 1) (54–63). All these clinical evaluations are coupled with the examination of cognitive functions (attention, memory, psychomotor speed, flexibility, etc.), standard biological data (complete blood count, electrolytes, hepatic enzymes, creatinine clearance, etc.), metabolic tests (glycemia, cholesterolemia, etc.), morning cortisol, thyroid function, and vitamin status (D, B9, B12, etc.).

### Feedback to the Clinicians

One of the major roles of the expert centers is to provide feedback to the clinicians regarding the proposals for care. These reports cover both diagnosis, clinical and treatment issues. As the evaluation is carried out by several different experts, the document sent to the clinicians is structured as the report of a multidisciplinary clinical meeting. In addition, the network intends to propose formalized recommendations on TRD for clinicians through joined sponsorship with the French Association of Biological Psychiatry and Neuropsychopharmacology.

### Biobanking

A biobank was gradually developed and implemented within the network of the expert centers for resistant depression. After collecting signed written informed consent, each included patient is sampled at the beginning of the first visit within the Center of Clinical Investigation working locally in close collaboration with each of the expert centers. Samples are then transferred and stored at the Mondor CRB (Créteil) for further research projects developed within the network.

Building this biobank should enable the identification of new biomarkers and precise mechanisms of resistance with the identification of immunological, neuroendocrine, biochemical, genetic, epigenetic and cellular mechanisms regulating the signaling pathways that are supposed to be impaired in resistant depression. For this purpose, the biobank is composed of serum, plasma, DNA, RNA, etc. to be able to measure several biomarkers associated with the immune (pro-inflammatory cytokines and enzymatic pathways), neuroendocrine (pituitary–adrenal and thyroid axes), and monoaminergic systems (serotonin, norepinephrine, dopamine), or implicated in neurogenesis, neuroprotection (e.g., BDNF), and in genetic/epigenetic mechanisms.

### Longitudinal Follow-up

**Figure 2:**
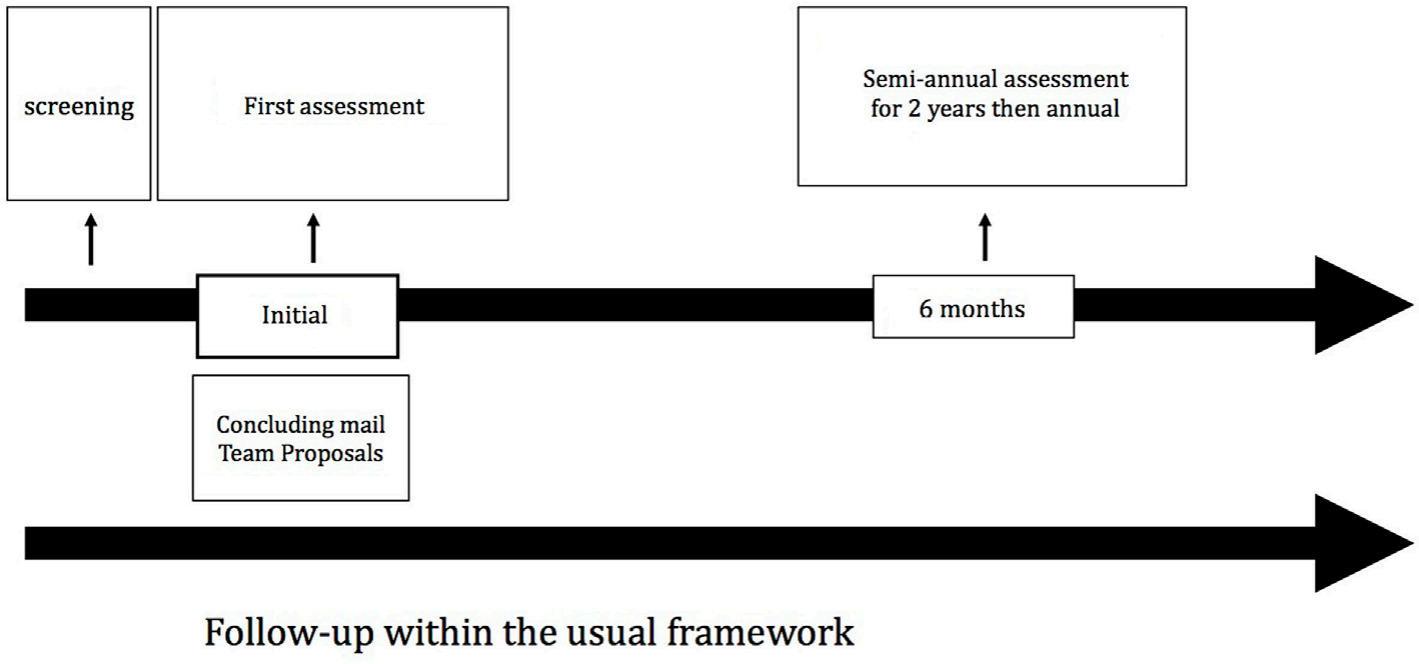
Follow-up of an individual patient within the French Treatment-Resistant Depression Network.

Each patient enrolled into the cohort is evaluated every six months over the next 4 years (Figure 2). Table 2 summarizes the evaluation tools used at each visit. The objective of these follow-up visits is to evaluate the course of depressive symptoms and related clinical dimensions such as clinical remission, residual symptoms, relapses, recurrences, etc.

**Table 2:** Standardized scales of assessment.

Assessment	Scale	Pre-screening	First assessment	Months 6 and 18	Months 12 and 36	Months 24 and 48
Current mood states and residual symptoms	– MINI	X				

	– Thase and Rush	X				

	– QIDS-SR16	X	X	X	X	X

	– MADRS	X	X	X	X	X

	– ROSENBERG scale – ALTMAN – MATHYS – STAI FORME YA – SHAPS – BAS – YMRS – Widlöcher		X	X	X	X

	– SPIN		X			X

	– SACHS		X			X

Mood state lifespan	– MDQ		X		X	X

Psychiatric comorbidities and trauma	– Barratt – Big Five Inventory – PCL-S – CTQ		X			X

Suicidal behavior	– ISF, SIS		X			X

	– C-SSRS		X	X	X	X

Sleep	– CSM – Epworth		X		X	X

	– PSQI		X	X	X	X

Functioning and severity of disorder	– FAST		X			X

	– CGI-S – EGF – ED-5Q-5L		X	X	X	X

	– CGI-I			X	X	X

Lifestyle	– Physical activity (QIAP) – Food Questionnaire – LEAPS – Amiel-Lebigre – Paykel		X		X	X

Care course	– CSRI		X		X	X

Treatment: Psychotropic Psychotherapy Brain stimulation	– MARS – PRISE-M – ATHF – PTHF – e-Resistant Depression		X	X	X	X

Cognitive functioning	– CLVT – WAIS – TMT A – TMT B – Stroop test – verbal Fluency		X		X	X

*BAS, Brief Anxiety Scale; CLVT, California Verbal Learning Test; CSM, composite scale of morningness; FAST, Functional Analysis Screening Tool; MDQ, Mood Disorder Questionnaire; PSQI, The Pittsburgh Sleep Quality Index; QIDS-SR16, Quick Inventory of Depressive Symptomatology self report; SHAPS, Snaith–Hamilton Pleasure Scale; SIS, Suicide Intent Scale; SPIN, Social Phobia Inventory; TMT A and B, Trail Making Test A and B; WAIS, Wechsler Adult Intelligence Scale; YMRS, Young Mania Rating Scale; MINI, Mini International Neuropsychiatric Interview; PCL-S, Post Traumatic Stress Disorder Check List Scale; PRISE-M, Patient Rated Inventory of Side Effects; SACHS, Bipolar Index; ATHF, Criteria for Rating Medication Trials for Antidepressant Failure; C-SSRS, Columbia-Suicide Severity Rating Scale; CSRI, Client Service Receipt Inventory; CTQ, Child Trauma Questionnaire; ISF, Measure of Suicidal Ideation; LEAPS, Lam Employment Absence and Productivity Scale; MARS, Medication Adherence Rating Scale*.

At the end of each visit, a report is sent to the doctor who referred the patient to the center. As described earlier, the results of the main evaluations are provided in these reports, and a clear diagnosis is given as well as a relevant proposal for therapeutic management. Therefore, from the overall clinical and paraclinical assessments carried out and in relation to recommendations of good practice, individualized treatment interventions that may include innovative therapeutic strategies such as brain stimulation techniques are considered.

### Analysis Plan

Data will be analyzed using SPSS version 22 (Armonk, NY, USA: IBM Corp.). As described elsewhere (7, 12, 64), means and SDs will be presented for continuous variables, and counts and percentages for discrete variables. Parametric analysis-of-variance methods will be used for the repeated comparisons of the overall symptom severity (i.e., MADRS, QIDS-SR-16 scores) over the 4-year follow-up period. *Post hoc* tests will be conducted to make pairwise comparisons. Chi-square tests will be applied for the comparisons of sociodemographic characteristics, and percentages of depressed patients showing satisfactory response (i.e., ≥50% decrease in MADRS or QIDS-SR-16 score from baseline), achieving remission (i.e., MADRS score ≤10 or QIDS-SR-16 score ≤5) or experiencing relapse (i.e., MADRS score ≥15 or QIDS-SR-16 score ≥11) across experimental visits. For the calculation of sample size, we formulate the hypothesis that the depressed patients in our sample will be considered as remitters in at least 35% of cases at mid-term after the proposed treatment strategies referring to standard international guidelines, thereby enabling to obtain the remission rate observed following a first-line antidepressant therapy in the earlier collaborative study STAR*D (7). This therefore contrasts with the remission rate of less than 15% which was reported after failure of more than two previous antidepressant treatments in this pivotal STAR*D trial (7). On this basis, the required number of subjects is calculated for a two-sided test with a risk α of 5% and a power 1-β of 95% leading to the inclusion of a total of 130 resistant depressed subjects in our cohort to allow the demonstration of a difference at least equal to 20% in the remission rate as compared with that found in the STAR* study. We will recruit 130 additional subjects in our cohort to take into account and cover any protocol deviations, dropouts, or missing data, etc.

### Dissemination Plan

The significant findings will be disseminated through following three distinct and complementary strategies: (1) publications in international scientific journals, (2) communications in congress, and (3) symposia and conference for health care providers under supervision of the principle investigators within each university hospital affiliated to our national network. Other specific actions will be developed enabling the targeted diffusion of the most relevant results and including multidisciplinary brainstorming sessions in the setting of a regional coordination of care for difficult-to-treat depressed patients, teaching activity for medical students and residents in psychiatry, continuing medical education for mental health professionals (general practitioners, psychiatrists, psychologists, nurses, etc.), and educational training during summer schools for master and Ph.D. students, postdoctoral fellows, especially. Finally, more general informative meetings will be regularly conducted by the expert centers, designed for the affected patients with their caregivers and commonly focused on the presentation of major clinical, therapeutic, and functional issues related to resistant depression.

## Discussion

By offering a systematic, complete, longitudinal, and multidimensional evaluation of each TRD case, using shared medical records containing common assessment tools exploring separately the psychiatric, non-psychiatric dimensions, metabolic functions, biological, and cognitive impairments, we aim at making accessible to all patients with a diagnosis of TRD, the most personalized and adapted treatment strategies possible. Indeed, it seems important to be able to apply the therapeutic guidelines to improve the prognosis of TRD. Guo et al. (65) showed an improvement in the management using measurement-based care for outpatients with moderate to severe major depression. Indeed, the responses as well as remission rates are higher, and adjustment of therapeutic doses of antidepressant is more straightforward in the measurement-based care group. As proposed in the expert centers, the administration of a wide range of clinical instruments and neuropsychological tests for the comprehensive assessment of depressed patients could be perceived negatively, especially with regard to the long time, attentional demand and sustained effort required for completion. However, clear information about the study design is delivered before final acceptance of participation. All these assessments are scheduled over three half-days on two consecutive days but interleaved systematically with frequent rest periods to prevent sufficiently a gradual decline in concentration and excessive fatigability. Phone reminders are even scheduled at least one week before each visit to limit dropouts. As supported in the study by Rush (66), measurement-based care also offers the advantage of better preparing and engaging the patients in shared decision making and adherence to long-term care needs, thereby familiarizing themselves with relevant tools currently used for monitoring their clinical symptoms and side effects caused by standard treatments.

Moreover, diagnostic and clinical heterogeneity appears to be a major obstacle for an exact understanding of the pathophysiology of mental illnesses and, in particular, major depression. Although there is a widely demonstrated hereditary component (67) the identification of genetic risk factors has proven difficult even in meta-analyses of genome-wide association studies (68).

Similarly, efforts to develop new treatments have stagnated, partly due to a lack of new biological targets and the choice of the people most likely to benefit from them (69). All these challenges have been attributed in part to the fact that our diagnostic system assigns a unique label to a syndrome that is not unitary but could be caused by distinct pathological processes, which would require different treatments. Indeed, major depression is a widespread disease presumably represented by a large number of patient subgroups that must be identified precisely to propose adapted therapeutic strategies.

Our national network must be part of the existing regional health care system. Although adherence to treatment guidelines has been shown to improve clinical outcomes (65), there are barriers to their application, including the fact that guidelines are often viewed as “top-down” measures. One aim of the network is to make available the standard recommendations derived from the currently used guidelines for each patient. It should bridge the gap between evidence-based medicine and routine practice.

In addition, clinical research is an important issue within the network. The electronic health-care record system for each expert center is connected to a national database and data on this large clinically representative cohort of TRD cases will allow prospective follow-up and comparative-effectiveness studies. Combination of clinical, biological, genetic, and epigenetic data could contribute to a more precise definition of separate clusters of major depression for a more specific and tailored management of this pathology. To refine the diagnostic and therapeutic approach, by focusing on more precise clusters, the main lines of future research are focused on: (i) the role of comorbidities, beyond what are now defined as risk factors for resistance [cardiovascular, neurological, metabolic pathologies, etc. (70–72)]; (ii) the role and impact of traumatic factors; (iii) the presence of suicidal ideation or behaviors; (iv) the functional repercussions; (v) resistance to treatments; (vi) neurocognitive profiles; (vii) genetic, epigenetic, immuno-inflammatory, neuroendocrine, and neurochemical markers, etc. In this context, patients benefiting from the evaluation within the expert centers, are asked to participate in various national and European research studies implemented in partnership with the “Fondation FondaMental” addressing jointly clinical, pathophysiological, and therapeutic issues (73).

## Conclusion

The adopted integrative approach shared within the network of expert centers for resistant depression is a compelling model for the precise characterization of sociodemographic, clinical, neuropsychological, biological, and metabolic factors of treatment resistance and to further develop innovative and personalized strategies through the multi-faceted questions of assessment, management, and treatment of major depression. This is one of the best options for changing the poor prognosis of TRD, reducing the deleterious impact on daily functioning and quality of life and thereby lowering the serious societal and economic burden related to high direct and indirect costs of TRD.

## Ethics Statement

Ethics approval and consent to participate: the database (clinical data and biobanking) was validated by an ethics committee: CNIL (French CNIL: DR-2015-673). Consent for publication: all patients in the expert centers receive oral and written information on the possible use of their data for research purposes. Availability of data and material: all the material used for the assessment is validated in French.

## Author Contributions

AY, DB, EH, WE–H, and BA realized the initial frame of the manuscript. The authors have made corrections after proofreading. All the authors participated in the recruitment of patients.

## Conflict of Interest Statement

AY: I acted in advisory capacities, carried out clinical studies in relation to the development of a medicine (Janssen), received travel allowance, gave presentations at meetings, and received remuneration for my input from the following pharmaceutical organizations: AstraZeneca, Janssen, Lundbeck, Otsuka, and Servier. DB has received honoraria from Lundbeck. EH: I acted in advisory capacities, carried out clinical studies in relation to the development of a medicine, received personal researches, studies or travel allowance, gave presentations at meetings, and received remuneration for my input from the following pharmaceutical organizations: AstraZeneca, BMS, Cellgene, Euthérapie—Servier, Janssen, Elli Lilly, Lundbeck, Otsuka, Pfizer, and Sanofi. And, I held a managerial position in Fondation FondaMental, Créteil, and the French Association of Biological Psychiatry. MG has received honoraria from Janssen—Cilag and Lundbeck. TD, PD, LM, and LS: no competing interest. OD has received honoraria from Lilly, AstraZeneca, Jansen, Servier, and Lundbeck. FH has received honoraria from AstraZeneca, a grant from Institut Servier and is currently supported by Fonds de recherche du Québec—Nature et technologies (grant number #200123.). FS has received honoraria from Otsuka. GV has received speaker honoraria from Otsuka/Lundbeck (annual intervention at the reception day for new professors of psychiatry). WE-H has received speaker honoraria from Janssen, Lundbeck, Otsuka, and UCB. BA: as received speaker honoraria from Lundbeck, Janssen, and Eli Lilly. All other authors declare that the research was conducted in the absence of any commercial or financial relationships that could be construed as a potential conflict of interest.

## Abbreviations

ATHF, Criteria for Rating Medication Trials for Antidepressant Failure; BAS, Brief Anxiety Scale; BFI, Big Five Inventory; CGI-S/I; Clinical Global Impression Severity/Improvement; CLVT, California Verbal Learning Test; C-SSRS, Columbia-Suicide Severity Rating Scale; CSM, composite scale of morningness; CSRI, Client Service Receipt Inventory; CTQ, Child Trauma Questionnaire; EQ-5D-5L, EuroQol Five Dimension Five Level; FAST, Functional Analysis Screening Tool; GAF, Global Assessment of Functioning; ISF, Measure of Suicidal Ideation; IPAQ, International Physical Activity Questionnaire; LEAPS, Lam Employment Absence and Productivity Scale; MARS, Medication Adherence Rating Scale; MDQ, Mood Disorder Questionnaire; MINI, Mini International Neuropsychiatric Interview; PCL-S, Post Traumatic Stress Disorder Check List Scale; PRISE-M, Patient Rated Inventory of Side Effects; PSQI, The Pittsburgh Sleep Quality Index; QIDS-SR16, Quick Inventory of Depressive Symptomatology self report 16; SACHS, Bipolar Index; SHAPS, Snaith–Hamilton Pleasure Scale; SIS, Suicide Intent Scale; SPIN, Social Phobia Inventory; STAI-A, State-Trait Anxiety Inventory A; TMT A and B, Trail Making Test A and B; TRD, treatment-resistant depression; WAIS, Wechsler Adult Intelligence Scale; YMRS, Young Mania Rating Scale.
